# Multiplex digital PCR: a superior technique to qPCR for the simultaneous detection of duck Tembusu virus, duck circovirus, and new duck reovirus

**DOI:** 10.3389/fvets.2023.1222789

**Published:** 2023-08-10

**Authors:** Yanwen Yin, Chenyong Xiong, Kaichuang Shi, Feng Long, Shuping Feng, Sujie Qu, Wenjun Lu, Meizhi Huang, Changhua Lin, Wenchao Sun, Zongqiang Li

**Affiliations:** ^1^Guangxi Center for Animal Disease Control and Prevention, Nanning, China; ^2^College of Animal Science and Technology, Guangxi University, Nanning, China; ^3^Longan Center for Animal Disease Control and Prevention, Nanning, China; ^4^Guangxi State Farms Yongxin Animal Husbandry Group Xijiang Co., Ltd., Guigang, China; ^5^Wenzhou Key Laboratory for Virology and Immunology, Institute of Virology, Wenzhou University, Wenzhou, China

**Keywords:** digital PCR, coefficients of variation, duck Tembusu virus, duck circovirus, new duck reovirus

## Abstract

Duck Tembusu virus (DTMUV), duck circovirus (DuCV), and new duck reovirus (NDRV) have seriously hindered the development of the poultry industry in China. To detect the three pathogens simultaneously, a multiplex digital PCR (dPCR) was developed and compared with multiplex qPCR in this study. The multiplex dPCR was able to specifically detect DTMUV, DuCV, and NDRV but not amplify Muscovy duck reovirus (MDRV), Muscovy duck parvovirus (MDPV), goose parvovirus (GPV), H4 avian influenza virus (H4 AIV), H6 avian influenza virus (H6 AIV), and Newcastle disease virus (NDV). The standard curves showed excellent linearity in multiplex dPCR and qPCR and were positively correlated. The sensitivity results showed that the lowest detection limit of multiplex dPCR was 1.3 copies/μL, which was 10 times higher than that of multiplex qPCR. The reproducibility results showed that the intra- and interassay coefficients of variation were 0.06–1.94%. A total of 173 clinical samples were tested to assess the usefulness of the method; the positive detection rates for DTMUV, DuCV, and NDRV were 18.5, 29.5, and 14.5%, respectively, which were approximately 4% higher than those of multiplex qPCR, and the kappa values for the clinical detection results of multiplex dPCR and qPCR were 0.85, 0.89, and 0.86, indicating that the two methods were in excellent agreement.

## Introduction

Tembusu virus (TMUV), a positive-sense single-stranded RNA virus belonging to the *Flaviviridae* family and *Flavivirus* genus (1), was first detected in the Malaysian Culex mosquito in 1955 and became endemic in duck farms in southeastern coastal China in 2010 (2, 3). DTMUV has a wide range of hosts, such as chickens, ducks, geese, pigeons, and sparrows, and the diseased ducks show a series of symptoms, including ovarian hemorrhage and necrosis, decreased egg production, ataxia, diarrhea, and enlarged spleen and liver, with an incidence of up to 100% and mortality rates between 5 and 30% (4, 5); moreover, younger ducks exhibit more serious pathogenicity (6). Reportedly, DTMUV antibodies have been detected in the serum of duck farm workers (7), indicating that the disease may pose a public safety risk. Duck circovirus (DuCV) is a circular single-stranded DNA virus belonging to the genus Circovirus of the family Circoviridae (8), which first appeared in Germany in 2003 and subsequently spread to China, Korea, and the United States (9, 10). DuCV is highly contagious, mainly through horizontal and vertical transmission (11, 12); ducks of all ages can be infected, and the highest rate of infection is observed 3 to 4 weeks of age (13). Moreover, the incidence of this virus is 10–81.6% in China, and the disease ducks show feathering disorders, stunted growth, reduced production performance, immune organ defects and cell necrosis (14). The abovementioned features seriously affect the development of the duck breeding industry. DuCV-1 and DuCV-2 are prevalent in China, but a recent study revealed the identification of a new genotype (DuCV3) in duck farms in Hunan, China (15), which has attracted attention. Novel duck reovirus (NDRV) belongs to the Orthoreovirus genus in the family Reoviridae and is a double-stranded RNA virus (16). NDRV was first identified in Fujian in 2005 (17) and has become endemic in duck farms in China. NDRV can cause morbidity in ducks and geese, and ducks are most susceptible at 7–14 days of age, with an incidence of 5–35% and mortality of 2–20% (18). Ducks exhibit a pathology characterized by hemorrhagic necrosis of the liver and spleen, diarrhea, and growth retardation, and the disease is also known as spleen necrosis disease or duck hemorrhagic-necrotic hepatitis (19). In summary, DTMUV, DuCV, and NDRV, which are commonly prevalent in the farming industry in China, have a great impact on and causes loss to the poultry farming industry. Therefore, early diagnosis and prevention of the spread of these pathogens in poultry flocks are of great practical importance.

Digital PCR (dPCR) is a nucleic acid absolute quantification technology developed in the late 1990s (20). This technique can be understood as single-molecule-level fluorescent PCR amplification in large-scale parallel microreactors, and after the PCR reaction, the copy number of the target nucleic acid sequence is calculated based on the fluorescent signal and Poisson distribution (21). Due to its advantages of high sensitivity, high accuracy, high tolerance, and absolute quantification (22), dPCR is superior to qPCR technology in clinical settings and has been widely used in genetic mutation diagnosis, copy number variation analysis, and pathogenic microorganism detection. In this study, a new method was developed by applying multiplex dPCR technology for the first time, and this developed method provides a technical tool for the simultaneous detection of DTMUV, DuCV, and NDRV.

## Materials and methods

### Viral strains and clinical samples

The viral strains included DTMUV (tissue sample), DuCV (tissue sample), NDRV (tissue sample), Muscovy duck reovirus (MDRV, tissue sample), Muscovy duck parvovirus (MDPV, tissue sample), goose parvovirus (GPV, tissue sample), H4 avian influenza virus (H4 AIV, tissue sample), H6 avian influenza virus (H6 AIV, tissue sample), and Newcastle disease virus (NDV, La Sota vaccine strain). A total of 173 clinical tissue samples of ducks were collected from Guangxi in China. The tissues were placed into 2.0-mL centrifuge tubes containing PBS at pH 7.0, ground into a crumbly form using a grinder, and centrifuged at 10,000 rpm for 5 min, and the supernatant was used for the extraction of total nucleic acids using a TaKaRa MiniBEST Viral RNA/DNA Extraction Kit Ver.5.0 (Dalian, China). RNA from clinical samples or vaccine strains was reverse transcribed to cDNA using the PrimeScript™ II 1st Strand cDNA Synthesis Kit (Dalian, China), and the DNA and cDNA were stored at −80°C. All clinical tissue samples were collected from sick and dead ducks transported from farms and stored at the Guangxi Animal Disease Prevention and Control Center in China.

### Primers and probes

Primers and probes for multiplex dPCR amplification of the DTMUV (KC990540) C gene, DuCV (MK814589) rep gene, and NDRV (GQ888710) S3 gene were designed using Olign 7.6 (Table 1).

**Table 1:** Primers and probes for multiplex dPCR amplification.

**Primers and probes**	**Sequence (5^′^ → 3^′^)**	**Product size/bp**
DTMUV-Fq	GGCCGGGTTGTCAATATGC	80
DTMUV-Rq	ACCCCATCAATCGTCCTCTTT	
DTMUV-P	FAM-AAAGCGCGGAACGTCCCGC-BHQ1	
DuCV-Fq	AGCCGTTATGCRTTTGAATTTC	75
DuCV-Rq	CGAGTAACCGTCCCACCACTT	
DuCV-P	VIC-CCGAAAACAAGTATTACAAACCACGCGG-BHQ1	
NDRV-Fq	GGGTCGCACTACAGAGCAACT	76
NDRV-Rq	CGCCTCATCATAGTAATCTGCAA	
NDRV-P	CY5-CTTGATCAATATGCCGTTGCTCTGCATG-BHQ3	

### Construction of standard plasmids

PCR amplification was performed with DTMUV, DuCV, NDRV cDNA, or DNA as a template using three pairs of specific primers, and the PCR products were purified by applying the TaKaRa MiniBEST Agarose Gel DNA Extraction Kit Ver.4.0 (Dalian, China) and ligated into the pMD™18-T Vector (China); the constructs were transformed into E. coli DH5α competent cells, coated and incubated at 37°C for 16 h, and the TaKaRa MiniBEST Plasmid Purification Kit Ver. 4.0 (Dalian, China) was applied for extraction of the plasmids, which were named p-DTMUV, p-DuCV, and p-NDRV, respectively, and sequencing. The concentration of each plasmid was determined and normalized using the following formula: plasmid copy number (copies/μL) = plasmid concentration × 10^−9^ × 6.02 × 10^23^)/(660 Dalton/bases × DNA length).

### Optimization of optimal reaction conditions for multiplex dPCR

The Naica^TM^ System (Stilla Technologies, Villejuif, France) was applied to optimize the DTMUV, DuCV, and NDRV primer and probe concentrations and annealing temperature (55–60°C). The reaction systems included 12.5 μL of PerfeCTa Multiplex qPCR ToughMix UNG (USA); 2.5 μL of Fluorescein Sodium Salt (Beijing, China); primers and probes for DTMUV, DuCV, and NDRV at concentrations of 600–1,000 nM and 200–400 nM, respectively; 2.5 μL of template; and double distilled water up to 25 μL. The reaction procedure was as follows: Step 1: predenaturation at 95°C for 5 min; Step 2: 45 cycles of denaturation at 95°C for 5 s, annealing at 57°C for 30 s, and 72°C for 30 s; Step 3: 72°C for 5 min. Moreover, the QuantStudio 5 qPCR detection system (USA) was applied to optimize the reaction conditions for qPCR.

### Construction of standard curves

Equal amounts of the standard plasmids for the three viruses were mixed and diluted to 1.3 × 10^5^-1.3 × 10^1^ copies/μL via 10-fold serial dilution. The linearity of multiplex dPCR and qPCR was statistically analyzed using SPSS 27.0, the correlation between them was assessed, and standard curves were plotted.

### Specificity, sensitivity, and reproducibility assays

Multiplex dPCR amplification was performed using DTMUV, DuCV, NDRV, MDRV, MDPV, GPV, H4 AIV, H6 AIV, and NDV cDNA or DNA as templates and sterilized water as a negative control to assess the specificity.

Equal amounts of standard plasmids for the three viruses were mixed, diluted to 1.3 × 10^8^ copies/μL−1.3 × 10^0^ copies/μL via 10-fold serial dilution and used as templates for multiplex dPCR and multiplex qPCR amplification to evaluate the detection limits.

The standard plasmids for the three viruses were mixed in equal volumes and diluted via 10-fold serial dilution to 1.3 × 10^5^ copies/μL, 1.3 × 10^4^ copies/μL, and 1.3 × 10^3^ copies/μL for use as templates for multiplex dPCR amplification to assess the reproducibility.

### Clinical assays for multiplex dPCR and multiplex qPCR

A total of 173 clinical samples were tested by multiplex dPCR to verify their utility. The reaction system consisted of 12.5 μL of PerfeCTa Multiplex qPCR ToughMix UNG (USA), 2.5 μL of Fluorescein Sodium Salt (Beijing, China), 0.9 μL each of DTMUV-Fq and DTMUV-Rq (25 pmol/μL), 0.3 μL of DTMUV-P (25 pmol/μL), 0.8 μL of DuCV-Fq and DuCV-Rq (25 pmol/μL), 0.3 μL of DuCV-P (25 pmol/μL), 0.9 μL each of NDRV-Fq and NDRV-Rq (25 pmol/μL), 0.3 μL of NDRV-P (25 pmol/μL), 2.5 μL of template, and double-distilled water to 25 μL. The reaction procedure was as follows: Step 1: pre-denaturation at 95°C for 5 min; Step 2: 45 cycles of denaturation at 95°C for 5 s, annealing at 57°C for 30 s, and 72°C for 30 s; Step 3: 72°C for 5 min (Table 2). Moreover, multiplex qPCR was applied to examine the same diseased material. Kappa values were calculated to compare the consistency of the two assays (when K < 0, the consistency strength was very poor; 0–0.2, weak; 0.21–0.4 weak; 0.41–0.6, moderate; 0.61–0.8, high; 0.81–1, very strong).

**Table 2:** Optimal reaction system for multiplex dPCR and multiplex qPCR.

**Reagents**	**Multiplex dPCR**		**Multiplex qPCR**	
	**Volume (**μ**L)**	**Final concentration (nM)**	**Volume (**μ**L)**	**Final concentration (nM)**
PerfeCTa multiplex qPCR ToughMix UNG	12.5	/	/	/
Premix Ex Taq™ (Probe qPCR)	/	/	12.5	/
Fluorescein sodium salt	2.5	/	/	/
DTMUV-Fq (25 μM)	0.9	900	0.1	100
DTMUV-Rq (25 μM)	0.9	900	0.1	100
DTMUV-P (25 μM)	0.3	300	0.1	100
DuCV-Fq (25 μM)	0.8	800	0.2	200
DuCV-Rq (25 μM)	0.8	800	0.2	200
DuCV-P (25 μM)	0.3	300	0.3	300
NDRV-Fq (25 μM)	0.9	900	0.2	200
NDRV-Rq (25 μM)	0.9	900	0.2	200
NDRV-P (25 μM)	0.3	300	0.2	200
Template	2.5	/	2.5	/
Double-distilled water	Up to 25	/	Up to 25	/

## Results

### Construction of standard plasmids

Standard plasmids for three viruses were obtained and named p-DTMUV, p-DuCV, and p-NDRV. The constructs were verified by sequencing. The concentrations were calculated using the formula and were found to equal 2.13 × 10^10^ copies/μL, 1.74 × 10^10^ copies/μL, and 1.30 × 10^10^ copies/μL, respectively, and the samples were diluted to obtain the same concentration of 1.30 × 10^10^ copies/μL.

### Optimal reaction conditions

**Figure 1:**
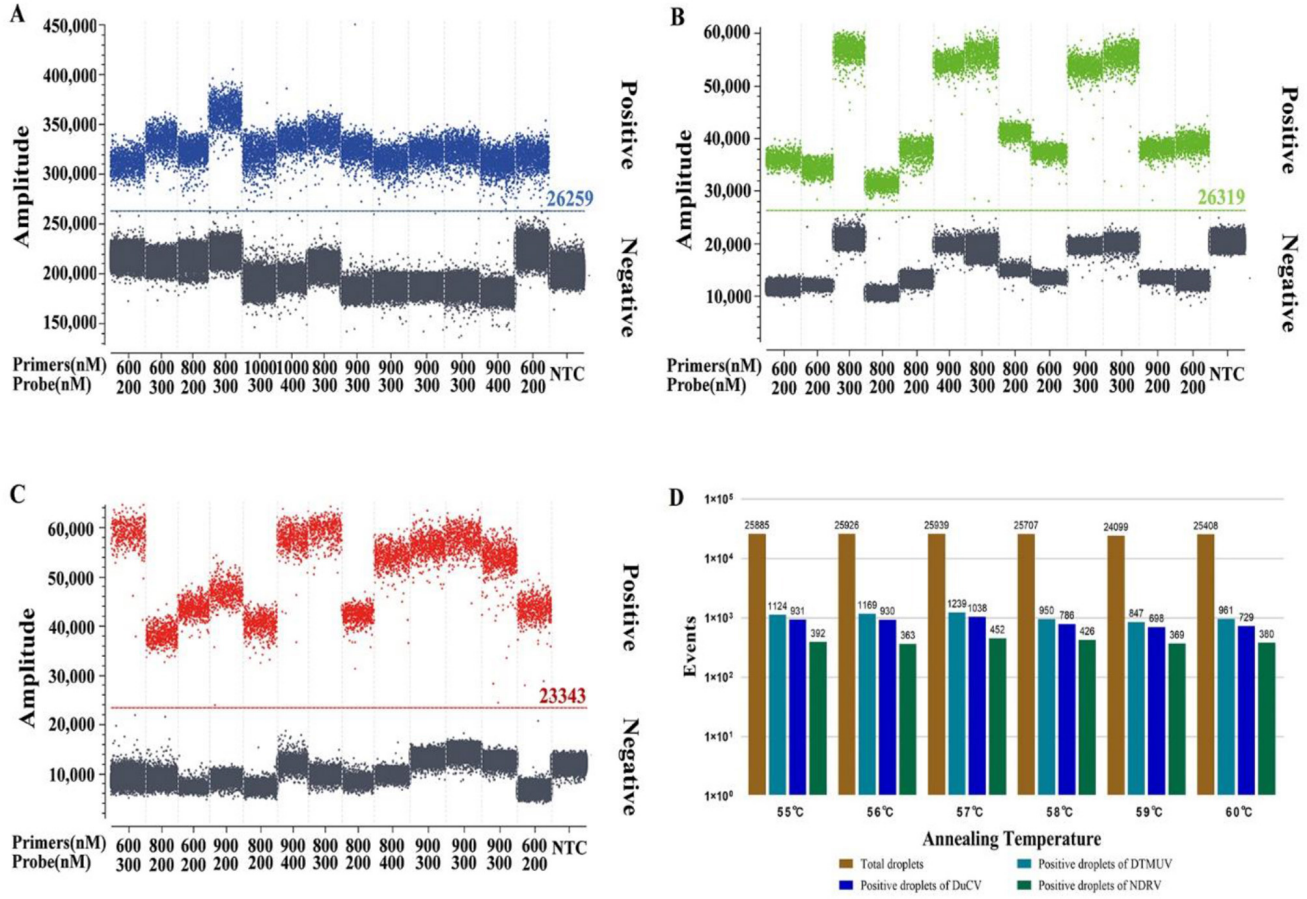
Optimization of the reaction conditions for multiplex dPCR. **(A–C)** show the optimization results for the multiplex dPCR primer and probe concentrations for duck Tembusu virus, duck circovirus, and new duck reovirus, respectively, and **(D)** shows the annealing temperature.

The concentration combined with the high number of microdroplets generated, high fluorescence signal value of positive microdroplets, good microdroplet density, the clear distinction between fluorescence signal value of negative and positive microdroplets, and low number of intermediate diffuse microdroplets were selected as the best reaction conditions. The optimal primer and probe volumes and concentrations for dPCR of DTMUV, DuCV, and NDRV were as follows: 0.9 μL each for DTMUV-Fq and DTMUV-Rq (25 pmol/μL), 0.3 μL for DTMUV-P (25 pmol/μL), 0.8 μL each for DuCV-Fq and DuCV-Rq (25 pmol/μL), 0.3 μL for DuCV-P (25 pmol/μL), 0.9 μL each for NDRV-Fq and NDRV-Rq (25 pmol/μL), and 0.3 μL for NDRV-P (25 pmol/μL). The optimal annealing temperature for the three viruses was 57°C, which produced the highest number of positive and total droplets (Figure 1). Meanwhile, the optimal primer and probe volumes and concentrations for qPCR were as follows: 0.1 μL each for DTMUV-Fq, DTMUV-Rq, and DTMUV-P (25 pmol/μL), 0.2 μL each for DuCV-Fq and DuCV-Rq (25 pmol/μL), 0.3 μL for DuCV-P (25 pmol/μL), and 0.2 μL each for NDRV-Fq, NDRV-Rq, and NDRV-P (25 pmol/μL). The annealing temperature was 57°C.

### Construction of standard curves

**Figure 2:**
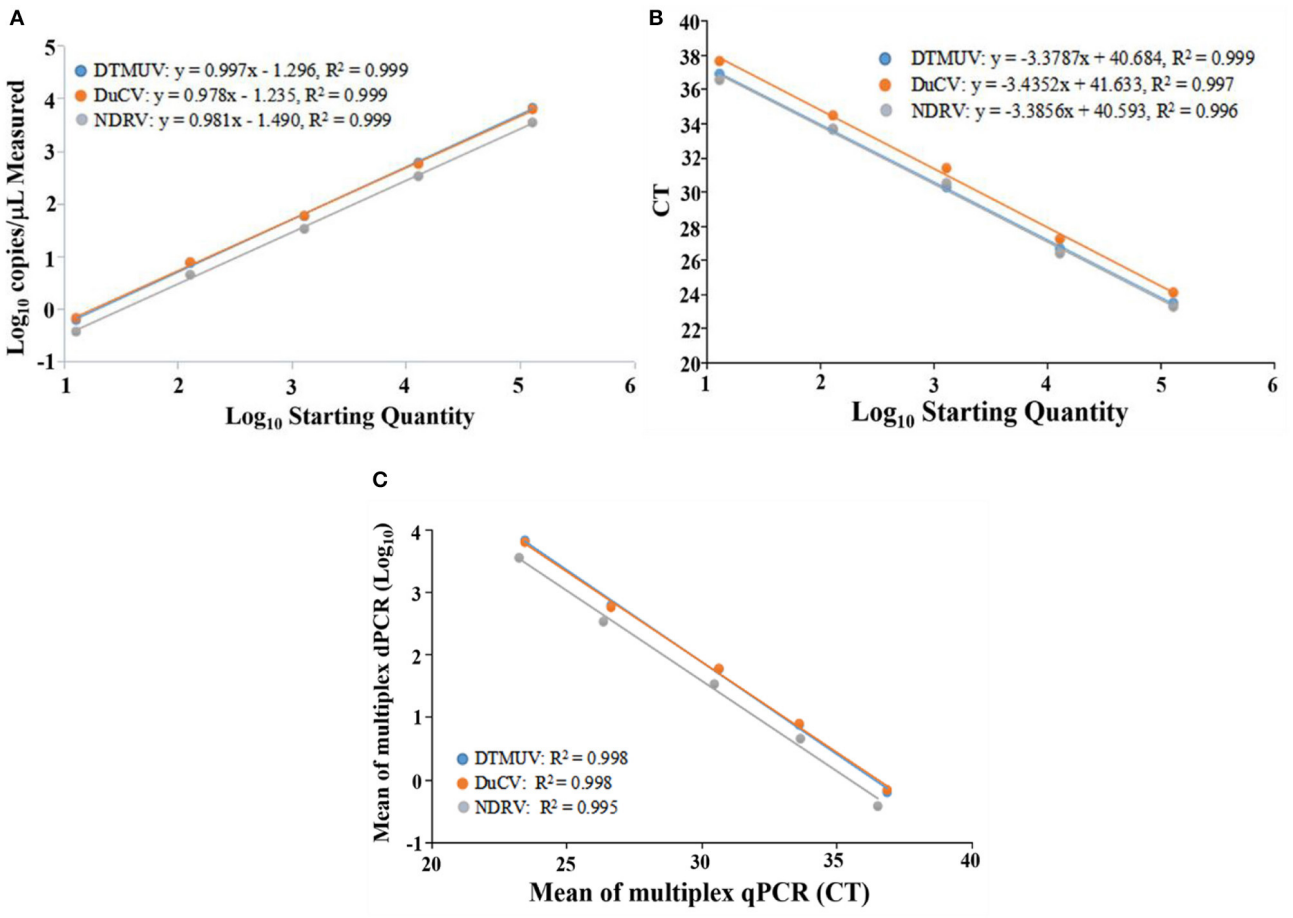
Standard curve of duck Tembusu virus, duck circovirus and new duck reovirus. **(A, B)** show the standard curves of multiplex dPCR and multiplex qPCR, respectively, and **(C)** indicates the correlation between them.

The multiplex dPCR standard curve results showed that the R^2^ and slope values for DTMUV, DuCV, and NDRV were −0.999 and 0.997, 0.999 and 0.978, and 0.999 and 0.981, respectively. The multiplex qPCR results showed that the R^2^ and slope values for DTMUV, DuCV, and NDRV were 0.999 and −3.3787, 0.997 and −3.4352, and 0.996 and −3.3856, respectively. The Pearson correlation coefficients for multiplex dPCR and qPCR for DTMUV, DuCV, and NDRV were 0.998, 0.998, and 0.995, respectively, indicating a positive correlation between the two methods (Figure 2).

### Analyses of specificity, sensitivity, and reproducibility

**Figure 3:**
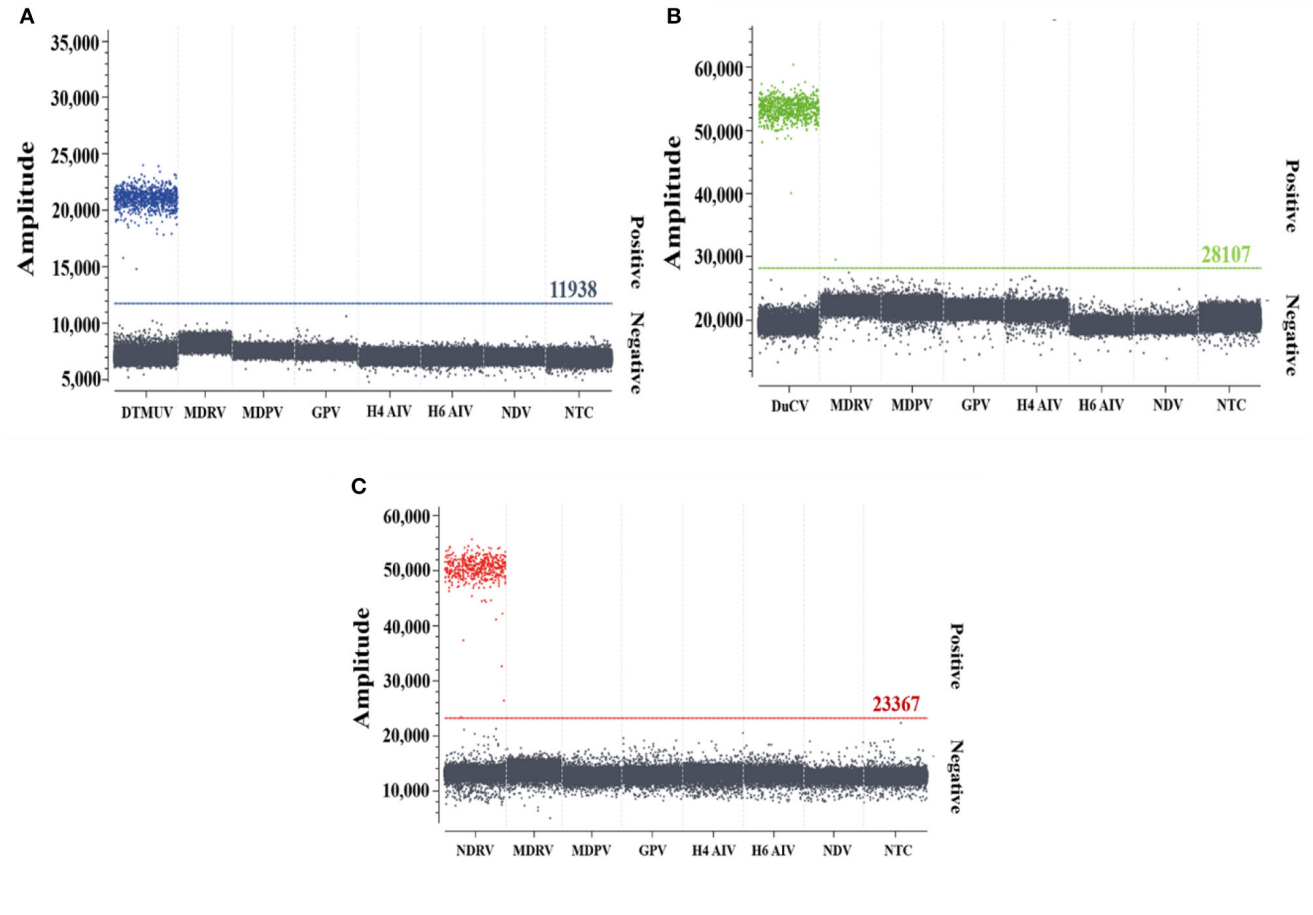
Specificity analysis of multiplex dPCR. **(A–C)** show the specific amplification of duck Tembusu virus, duck circovirus, and novel duck reovirus by multiplex dPCR, respectively.

**Figure 4:**
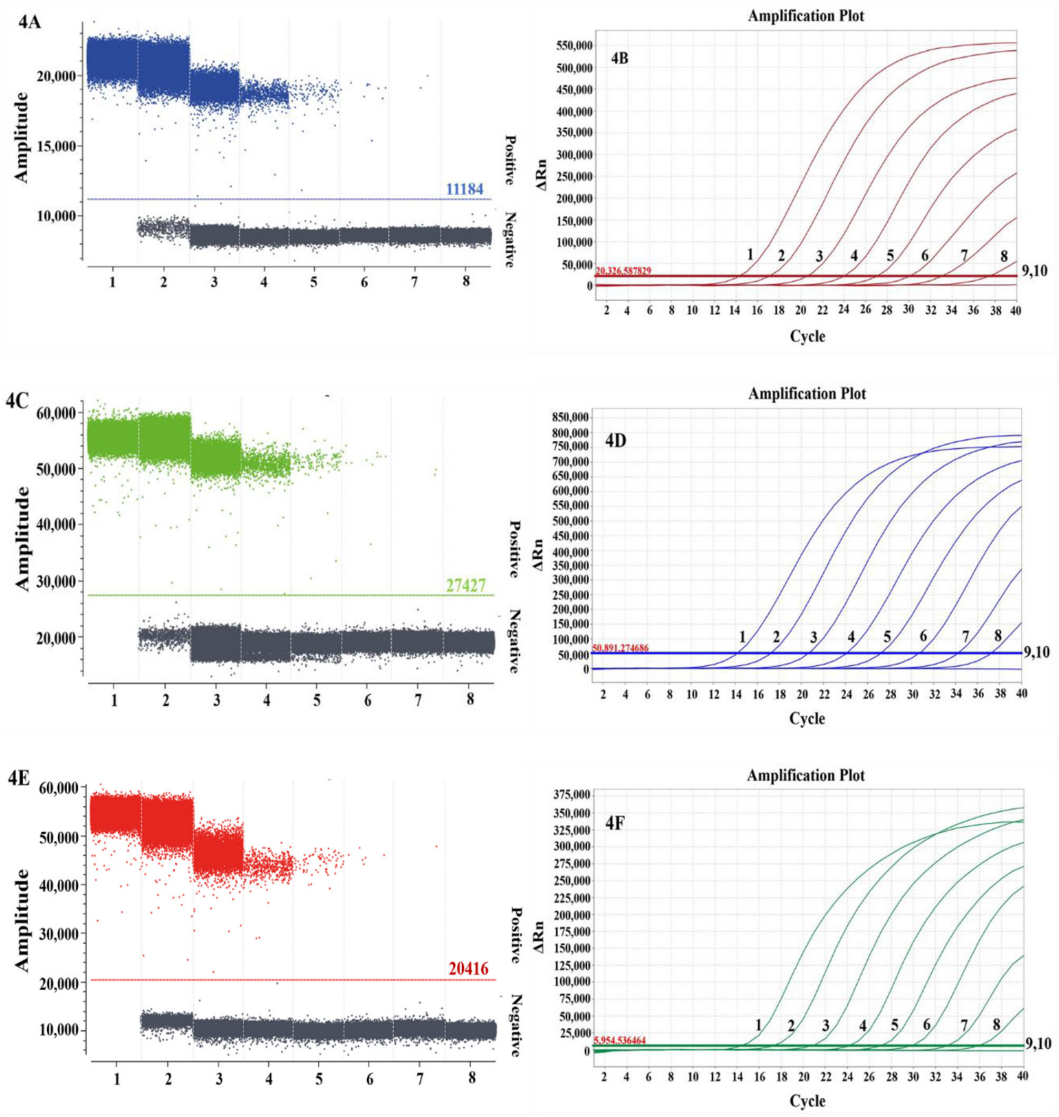
Comparison of the sensitivities of multiplex dPCR **(A, C, E)** and multiplex qPCR **(B, D, F)** for duck Tembusu virus **(A, B)**, duck circovirus **(C, D)**, and novel duck reovirus **(E, F)**. In **(A, C, E)**, 1–7 represent 1.30 × 10^6^-1.30 × 10^0^ copies/μL, and 8 represents the negative control; in **(B, D, F)**, 1–9 represent 1.30 × 10^8^-1.30 × 10^0^ copies/μL, and 10 represents the negative control.

The specificity analysis showed that only DTMUV, DuCV, and NDRV cDNA and DNA showed specific amplification, whereas MDRV, MDPV, GPV, H4 AIV, H6 AIV, and NDV cDNA and DNA and the negative control did not show positive microdroplets (Figure 3). The limit of detection for multiplex dPCR was 1.3 copies/μL, and that for multiplex qPCR was 13 copies/μL (Figure 4). The intra-assay coefficient of variation ranged from 0.06 to 1.35%, and the inter-assay coefficient of variation ranged from 0.23 to 1.94% (Table 3).

**Table 3:** Analysis of the repeatability of multiplex dPCR.

**Plasmid standards**	**Concentration (copies/μL)**	**Intra-assay repeatability**				**Interassay reproducibility**			
		**Measured values (copies/**μ**L)**			**CV (%)**	**Measured values (copies/**μ**L)**			**CV (%)**
p-DTMUV	1.3 × 10^5^	6,673	6,665	6,668	0.06	6,676	6,695	6,664	0.23
	1.3 × 10^4^	605.9	607.1	606.6	0.10	606.2	623.0	610.4	1.43
	1.3 × 10^3^	56.5	58.0	57.6	1.35	56.6	55.8	55.4	1.09
p-DuCV	1.3 × 10^5^	6,190	6,166	6,171	0.21	6,192	6,171	6,155	0.30
	1.3 × 10^4^	567.3	563.9	568.9	0.45	567.5	588.3	570.8	1.94
	1.3 × 10^3^	59.3	59.2	59.1	0.17	59.3	59.0	58.0	1.16
p-NDRV	1.3 × 10^5^	3,489	3,483	3,478	0.16	3,490	3,485	3,462	0.43
	1.3 × 10^4^	330.2	328.5	331.1	0.40	330.5	334.8	330.7	0.73
	1.3 × 10^3^	33.3	32.9	33.4	0.80	33.5	34.0	33.4	0.96

### Clinical results for multiplex dPCR and multiplex qPCR

A total of 173 clinical samples were tested by multiplex dPCR, and the results showed that the positive detection rates for DTMUV, DuCV, and NDRV were 18.5, 29.5, and 14.5%, respectively, and that there were cases of simultaneous infection by several viruses in the same clinical sample during the testing process. The mixed infection rates for DTMUV+DuCV, DTMUV+NDRV, DuCV+NDRV, and DTMUV+DuCV+NDRV were 5.2% (9/173), 2.9% (5/173), 1.7% (3/173), and 1.2% (2/173), respectively. Meanwhile, multiplex qPCR was used to probe the same diseased material; the DTMUV, DuCV, and NDRV positivity rates were 14.5, 25.4, and 11.0% (Table 4), respectively, and the coinfection rates for DTMUV+DuCV, DTMUV+NDRV, DuCV+NDRV, and DTMUV+DuCV+NDRV were 4.6% (8/173), 1.7% (3/173), 1.7% (3/173), and 1.2% (2/173), respectively. When the results of the two assays were compared, the compliance rates for DTMUV, DuCV, and NDRV were 96, 96, and 97%, respectively, and the kappa values for DTMUV, DuCV, and NDRV were 0.85, 0.89, and 0.86, respectively (Table 5).

**Table 4:** Clinical test results by multiplex dPCR and multiplex qPCR.

**Data**	**Number**	**Number of positive samples by multiplex dPCR**			**Number of positive samples by multiplex qPCR**		
		**DTMUV**	**DuCV**	**NDRV**	**DTMUV**	**DuCV**	**NDRV**
Aug 2020	57	1	17	3	1	13	2
Mar 2021	18	12	5	6	9	5	5
Apr 2021	33	14	9	0	12	9	0
Dec 2021	7	0	2	3	0	2	3
Mar 2022	48	3	8	13	1	8	9
Oct 2022	10	2	10	0	2	7	0
Total	173	32	51	25	25	44	19
Positivity rate %		18.5	29.5	14.5	14.5	25.4	11.0

**Table 5:** Analysis of the consistency of multiplex dPCR and multiplex qPCR.

**Detection method**	**Detection results (positive samples/total samples)**		
	**DTMUV**	**DuCV**	**NDRV**
Multiplex dPCR	32/173	51/173	25/173
Multiplex qPCR	25/173	44/173	19/173
Coincidence rate	96%	96%	97%
Kappa	0.85	0.89	0.86

## Discussion

In 2010, an infectious disease characterized by a sudden decrease in egg production (20–60%) emerged in Zhejiang, Fujian, Guangdong, and Jiangsu in China, and the causative virus was later confirmed to be DTMUV (23, 24). In autumn 2019, a new highly pathogenic DTMUV strain emerged in Anhui, China, belonging to genotype 2.2.1, which is far more pathogenic to geese and ducks than genotype 2.2 and may become the dominant group for DTMUV transmission in China (25). Epidemiological investigations have shown that DTMUV is widely prevalent in Chinese duck breeding farms, and DTMUV, AIV, duck hepatitis A virus, duck distemper virus, GPV, NDV, DuCV, fowl adenovirus, and Avian pathogenic Escherichia coli are the main pathogens that endanger the development of duck breeding industry and are often isolated from sick and dead ducks with serious mixed infections (26–28). The above findings show that DTMUV poses an extremely serious threat to China's waterfowl farming industry, and the infection is particularly complex and not easy to identify, requiring continuous epidemiological investigation and enhanced vaccination.

DuCV is an immunosuppressive virus that mainly affects the growth and development of ducks, attacks the immune system, aggravates the clinical symptoms of sick ducks and increases the mortality of sick ducks. When the immunity of the organism is reduced, opportunistic pathogens attack the ducks, and mixed and secondary infections occur. In China, DuCV has become prevalent, an incidences of infection have been reported in Guangxi (34.38%), Guangdong (25.6%), Yunnan (43.09%), and Shandong (33.29%) (8, 29, 30), with a large number of mixed infections with DuCV and *Riemerella anatipestifer, Escherichia coli* and duck hepatitis virus detected in duck farms in Shandong, at 8.28% (41/495), 4.85% (24/495), and 7.47% (37/495) (30), respectively. Similar coinfection with DuCV and other bacteria was detected in Korean duck flocks (31). In addition, we noted that mixed infections with DuCV and FAdV-4 may promote viral replication and cause more severe immunosuppression and injury (32). The spread of DuCV in China has caused considerable economic losses, and studies have suggested that migrating wild birds may play an important role in the spread, evolution and reorganization of DuCV (33), which is one of the reasons for the high incidence in China. Coupled with the lack of vaccines, the prevention of DuCV poses a serious challenge, and the key lies in strict testing of ducklings and elimination of positive ducks when introducing ducklings, strengthening feeding management, and improving environmental hygiene.

NDRV infection is an emerging infectious disease with clinical signs extremely similar to those of MDRV, but NDRV is more pathogenic. NDRV can cause immunosuppression, which leads to secondary and multiple infections (34), and most infected ducks die within 72 h (19). It is well known that most bacteria or viruses that attack animals cause damage to the spleen, liver, kidneys, and other organs of the host, thus exhibiting clinically similar symptoms and making it impossible to identify the pathogenic species. Early detection of the prevalence of the above pathogens in duck flocks can help interrupt the spread of the virus and reduce economic losses.

Due to the complexity of pathogenic infections and the immunosuppressive properties of DuCV and NDRV, multiple infections often occur with other viruses or bacteria, which are not easy to diagnose and differentiate. dPCR is a new tool for pathogen detection in the laboratory, has the advantages of absolute quantification without relying on standard curves, higher accuracy, and higher sensitivity, and has been used in medical and microbiological research (35). In this study, we developed a multiplex dPCR for the simultaneous detection of DTMUV, DuCV, and NDRV by optimizing the primers, probe concentration, and annealing temperature; the lowest detection limit reached 1.3 copies/μL, but that of the qPCR method established in this study was only 13 copies/μL; in addition, some qPCRs developed for DTMUV, DuCV, and NDRV have lower limits of detection of 10^1^-10^2^ copies/μL (36–40), which indicates that the sensitivity of our developed dPCR method has been further improved and is more suitable for the detection of pathogens at low concentrations. We also noted that the lower limits of detection of dPCR were 1 copies/μL−25 copies/μL regarding porcine circovirus type 2 (PCV2), porcine circovirus type 3 (PCV3), African swine fever virus (ASFV), classical swine fever virus (CSFV), porcine reproductive and respiratory syndrome (PRRS), and bovine leukemia virus (BLV) (41–44), indicating that the dPCR method we developed still has some advantages. We detected 173 clinical samples by dPCR, and the positive detection rates of DTMUV, DuCV, and NDRV were higher than those of qPCR, further validating the high sensitivity of dPCR. In addition, the established dPCR method is more precise than qPCR and can directly respond to the concentration of DTMUV, DuCV, and NDRV in clinical testing. However, despite the excellent detection sensitivity of dPCR, the clinical applications remain limited for various reasons, such as the limitations of the Naica^TM^ System, the low volume of the established dPCR method, the difficulty in carrying out pathogen detection on a large scale, and more expensive instrumentation and reagents. Compared with qPCR, the reaction mixture volume is limited, the operation is more tedious, and the risk of contamination is higher; thus, qPCR technology is still the most widely used genetic testing tool. With the update of science and technology, it is believed that these drawbacks of dPCR will be solved, and this method will thus be widely promoted in the field of microbiological research.

In conclusion, we developed a more sensitive multiplex dPCR for the simultaneous detection of DTMUV, DuCV, and NDRV, and this method provides a new tool for microbiological research.

## Data availability statement

The original contributions presented in the study are included in the article/supplementary material, further inquiries can be directed to the corresponding authors.

## Ethics statement

The animal study was reviewed and approved by Guangxi Center for Animal Disease Control and Prevention. Written informed consent was obtained from the owners for the participation of their animals in this study.

## Author contributions

YY and CX designed the research. KS and FL performed the majority of the experiments and were involved in preparation of the manuscript. SF, SQ, WL, MH, and CL analyzed data. WS and ZL revised the manuscript. All authors contributed to the article and approved the submitted version.
